# Advanced Current Collectors with Carbon Nanofoams for Electrochemically Stable Lithium—Sulfur Cells

**DOI:** 10.3390/nano11082083

**Published:** 2021-08-17

**Authors:** Shu-Yu Chen, Sheng-Heng Chung

**Affiliations:** 1 Department of Materials Science and Engineering, National Cheng Kung University, No. 1, University Road, Tainan City 701, Taiwan; f64071203@gs.ncku.edu.tw; 2 Hierarchical Green-Energy Materials Research Center, National Cheng Kung University, No. 1, University Road, Tainan City 701, Taiwan

**Keywords:** lithium–sulfur batteries, sulfur cathode, carbon nanofoam, graphene, MoS_2_

## Abstract

An inexpensive sulfur cathode with the highest possible charge storage capacity is attractive for the design of lithium-ion batteries with a high energy density and low cost. To promote existing lithium–sulfur battery technologies in the current energy storage market, it is critical to increase the electrochemical stability of the conversion-type sulfur cathode. Here, we present the adoption of a carbon nanofoam as an advanced current collector for the lithium–sulfur battery cathode. The carbon nanofoam has a conductive and tortuous network, which improves the conductivity of the sulfur cathode and reduces the loss of active material. The carbon nanofoam cathode thus enables the development of a high-loading sulfur cathode (4.8 mg cm^−2^) with a high discharge capacity that approaches 500 mA·h g^−1^ at the C/10 rate and an excellent cycle stability that achieves 90% capacity retention over 100 cycles. After adopting such an optimal cathode configuration, we superficially coat the carbon nanofoam with graphene and molybdenum disulfide (MoS_2_) to amplify the fast charge transfer and strong polysulfide-trapping capabilities, respectively. The highest charge storage capacity realized by the graphene-coated carbon nanofoam is 672 mA·h g^−1^ at the C/10 rate. The MoS_2_-coated carbon nanofoam features high electrochemical utilization attaining the high discharge capacity of 633 mA·h g^−1^ at the C/10 rate and stable cyclability featuring a capacity retention approaching 90%.

## 1. Introduction

The increasing demand for an advanced energy storage system has attracted the attention of researchers and inventors, who have investigated the possibility of next-generation rechargeable batteries with a high energy density and low cost [1,2,3]. Manganese (or nickel)-rich lithium nickel manganese cobalt oxide cathodes and sulfur cathodes are the most promising candidates, with sulfur having the highest charge storage capacity of 1672 mA·h g^−1^ and the lowest cost among all solid-state cathode materials [4,5,6]. Moreover, electrochemical conversion of the sulfur cathode occurs reversibly at a safe operating voltage of ~2.1 V and, most importantly, bypasses the limitation imposed by the crystalline structure of commercial insertion-type lithium-ion battery cathodes [5,6,7,8]. However, the conversion battery chemistry of lithium–sulfur batteries involves the repeated interconversion of the active material between solid and liquid states, along with the formation of new sulfur-based compounds [5,9,10,11]. At the full charge and discharge stages, the insulating sulfur and sulfides are deposited at the sulfur cathode. The resulting high cathode resistance makes it difficult for a sulfur cathode to effectively and reversibly utilize its high charge storage capacity [9,10,11]. During the discharge/charge processes, solid-state sulfur and sulfides convert to liquid-state lithium polysulfides (Li_2_S*_x_*, 4 ≤ *x* ≤ 8). The polysulfides are highly soluble in ether-based liquid electrolytes. Upon dissolution, the polysulfides irreversibly diffuse out of the cathode and uncontrollably migrate across the whole cell, which deteriorates both the electrodes and the electrolyte. Such polysulfide diffusion causes the capacity to fade quickly, resulting in a short cycle life [11,12,13].

To address these scientific issues, mainstream lithium–sulfur technologies are currently focused on optimizing the sulfur cathode [14,15,16] by the addition of conductive additives for high electrochemical utilization [14,15,16,17,18,19,20,21,22,23] and by the inclusion of porous substrates for strong polysulfide stabilization [14,15,16,20,21,22,23,24,25]. One of the most promising methods is the synthesis of sulfur-based nanocomposites that have various conductive and porous substrates and can easily form composites with sulfur. The resulting sulfur-based nanocomposite is subsequently coated onto an aluminum foil current collector by using additional carbon black and binder, followed by extra cathode preparation steps to further optimize cathode conductivity and integrity [14,15,16]. However, sulfur cathode chemistry dictates that the polysulfides that form during the intermediate charge and discharge stages inevitably diffuse out of the flat aluminum foil, eventually causing the loss of active material and damaging the integrity of the cathode. Moreover, the redeposition of the diffusing polysulfides in due course covers the two-dimensional cathode and hastens cathode failure [10,11,12]. To avoid this effect, the use of a porous current collector in the sulfur cathode has been proposed. A porous current collector is usually a conductive porous substrate with a conductive network that can transfer electrons throughout the cathode region [12,13,14]. Its porous structure ensures good electrolyte wetting and penetration, hosts the active material, and slows down the diffusion of polysulfides [26,27,28,29].

Here, we present a carbon nanofoam substrate as a porous current collector that features a carbon nanofiber skeleton with attached nanoporous carbon clusters. This conductive and porous carbon nanofoam substrate enables high electrochemical utilization and stability of the large amount of hosted sulfur [12,13,14,26,27,28]. The carbon nanofoam current collector enables a high sulfur loading of 4.8 mg cm^−2^ in the cathode, and stabilizes the high-loading sulfur cathode with a high charge storage capacity of 490–452 mA·h g^−1^ for 100 continuous cycles, indicating an excellent capacity retention of 90%. Inspired by these features, we further amplify the material characteristics by modifying the carbon nanofoam with graphene and molybdenum disulfide (MoS_2_) coatings to boost the charge transfer and polysulfide-trapping capabilities, respectively [17,18,19,30,31,32]. The modified carbon nanofoam enables the high-loading sulfur cathode to attain a high charge- storage capacity of 672 mA·h g^−1^ at the C/10 rate in the graphene-coated carbon nanofoam. The cathode simultaneously achieves high electrochemical utilization and stability in the MoS_2_-coated carbon nanofoam at a low electrolyte-to-sulfur ratio of 10 µL mg^−1^. Thus, in this study, we successfully demonstrate a cell configuration modification with a carbon nanofoam current collector and optimize it with a functional coating.

## 2. Materials and Methods

### 2.1. Materials and Chemical Characterization

Carbon nanofoam was obtained as commercial carbon paper (High Tech Material Solutions). The graphene-coated and MoS_2_-coated carbon nanofoams were prepared by chemical vapor deposition, using carbon nanofoam as the substrate. The graphene-coated carbon nanofoam was heated under argon gas at 1500 sccm, hydrogen gas at 200 sccm, and methane (CH_4_) at 5 sccm at 900 °C for 150 min. The MoS_2_-coated carbon nanofoam was prepared by depositing a thin film of molybdic acid (MoO_3_) on the carbon nanofoam at 300 °C and 2 × 10^−5^ torr with an e-beam at 0.5 A s^−1^. Subsequently, the MoO_3_-coated carbon nanofoam was treated under hydrogen sulfide (H_2_S) at 150 torr and 700 °C for an additional 60 min. The morphology, microstructure, and elemental analyses of the various carbon nanofoams were performed under a field emission scanning electron microscope (SEM) (SU-8000, Hitachi, Tokyo, Japan) and an energy-dispersive X-ray spectroscopy (EDX) spectrometer (XFlash 5010, Bruker, Billerica, MA, USA). The porosity analysis was performed by nitrogen adsorption–desorption isotherms at −196 °C using an automated gas sorption instrument (Autosorb iQ MP/MP, Anton Paar, Austria). The specific surface area and pore volume and size were calculated by the Brunauer–Emmett–Teller (BET) method with a 7-point model and subsequently confirmed by the Barrett–Joyner–Halenda (BJH), density functional theory (DFT), and Horvath–Kawazoe (HK) methods. Raman microscopy was performed using a Micro-Raman and Micro-PL spectrometer (Labram HR, Jobin Yvon, Paris, France) at 514 nm laser excitation.

### 2.2. Electrochemical and Cell Performance Characterization 

The carbon nanofoam and its two derivatives were used as porous current collectors to develop high-loading sulfur cathodes. The sulfur cathode was prepared by adding 25 µL of 1.0 M lithium polysulfide (Li_2_S_6_) catholyte into the carbon nanofoam current collector at a fixed sulfur loading of 4.8 mg cm^−2^. The Li_2_S_6_ catholyte was prepared by mixing stoichiometric amounts of sulfur (99.5%; Alfa Aesar, MA, USA) and lithium sulfide (Li_2_S; 99.9%; Alfa Aesar, MA, USA) with 1.85 M bis(trifluoromethane)sulfonimide lithium salt (99.95%; Sigma-Aldrich Corporation, MO, USA) and 0.1 M lithium nitrate co-salt (99.98%; Alfa Aesar, MA, USA) in a 1,3-dioxolane (99+%; Alfa Aesar, MA, USA) and 1,2-dimethoxyethane (99+%; Alfa Aesar, MA, USA) mixture. The high-loading sulfur cathode, polymeric separator, and lithium anode (99.9%; Sigma-Aldrich Corporation, MO, USA) were assembled in a coin cell with a low electrolyte-to-sulfur ratio of 10 µL mg^−1^. The electrochemical impedance spectra and cyclic voltammograms were recorded using integrated electrochemical workstations (SP-150 and VMP-300, Biologic, France) from 1 MHz to 100 mHz with an alternating-current voltage amplitude of 5 mV and set between 1.5 and 3.0 V at a scanning rate of 0.02 mV s^−1^, respectively. The discharge/charge voltage profiles and cyclability data were collected using a programmable battery cycler (BCS-800 series, Biologic, France) in a voltage window of 1.6–2.8 V at the C/10 rate. The current density of C/10 rate was calculated based on the mass loading and the theoretical charge storage capacity of sulfur (i.e., 1C = 1675 mA g^−1^).

## 3. Results and Discussion

### 3.1. Material Characterization of the Carbon Nanofoams

**Figure 1:**
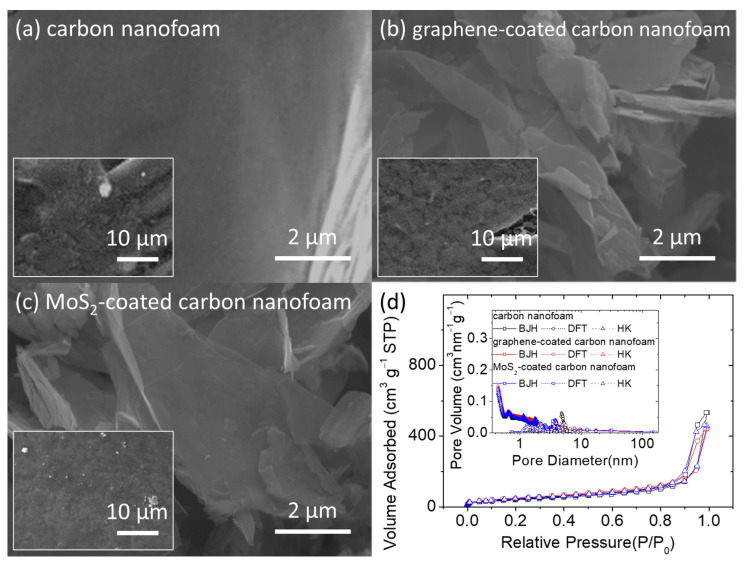
Material characterization: microstructural inspection of (**a**) carbon nanofoam, (**b**) graphene-coated carbon nanofoam, and (**c**) MoS_2_-coated carbon nanofoam. (**d**) Nitrogen adsorption–desorption isotherms; inset shows pore analysis by adsorption.

Figure 1 presents the microstructural inspection and porosity analysis of the unmodified, graphene-coated, and MoS_2_-coated carbon nanofoams. In the scanning electron microscopy (SEM) images, the unmodified carbon nanofoam appears to have a rough surface composed of nanoporous carbon clusters, with a carbon nanofiber skeleton that supports the continuous conductive network (Figure 1a). This structure enables the carbon nanofoam to host the insulating solid-state active materials and liquid-state polysulfides within the porous spaces of its conductive matrix [26,27,28]. The hosted active materials then possess smooth charge transfer capabilities and high material stability in the cathode [10,13]. The two main features of the carbon nanofoam, i.e., the conductive matrix that improves the reaction kinetics of sulfur [14,15,16,17,18,19,20,21,22,23] and the porous network that enhances the electrochemical stability of the polysulfides [14,15,16,20,21,22,23,24,25,26,27,28], are optimized by surface coating the carbon nanofoam with a layer of functional coating through chemical vapor deposition. This results in the formation of the graphene-coated and MoS_2_-coated carbon nanofoams that have a nanocoating attached on the carbon nanofoam substrates (Figure 1b,c).

Figure 1d shows a summary of the porosity analysis of the carbon nanofoams. The adsorption–desorption isotherms indicate that the nanoporous structures of the carbon nanofoams both with and without the surface coatings are similar. The carbon nanofoams show a microporous adsorption behavior at low relative pressure and a mesoporous adsorption–desorption behavior featuring the H3 loop. The H3-type hysteresis loop is shown by sheet-like materials (i.e., carbon nanofoam) and slit-shaped porous materials (i.e., graphene and MoS_2_ coatings). Pore analysis by adsorption and corresponding pore size distribution analysis from 0.5 to 160 nm are performed using the Barrett–Joyner–Halenda (BJH), density functional theory (DFT), and Horvath–Kawazoe (HK) methods. The pore analysis indicates that the nanopores of the carbon nanofoams are almost identical, which is consistent with the isotherms and clearly describes the microporous and mesoporous structures of the carbon substrate. The Brunauer–Emmett–Teller (BET) method is subsequently used to measure the specific surface area of the carbon nanofoams. The specific surface areas (with the total pore volume and average pore size in parentheses) of the unmodified, graphene-coated, and MoS_2_-coated carbon nanofoams are 145.8 m^2^ g^−1^ (0.8 cm^3^ g^−1^ and 3.8 nm), 164.6 m^2^ g^−1^ (0.7 cm^3^ g^−1^ and 3.4 nm), and 170.9 m^2^ g^−1^ (0.7 cm^3^ g^−1^ and 3.4 nm), respectively. The detailed analytical results indicate that after surface coating, the specific surface area increases, but the pore volume and pore size decrease. Moreover, the graphene-coated and MoS_2_-coated carbon nanofoams display similar nanopore characteristics. The porosity analysis of the carbon nanofoams therefore demonstrates that the appropriate modification of the carbon nanofoam could generate surface graphene and MoS_2_ coatings. It is possible that the coating layer covers the carbon nanofoam surface and slightly decreases the porosity. The functional coating consists of layered materials with high specific surface areas, which increases the specific surface area by modifying the matrix and incorporating the slit pores of the layered structure [14,15,16,23,24,25,26,27,28].

**Figure 2:**
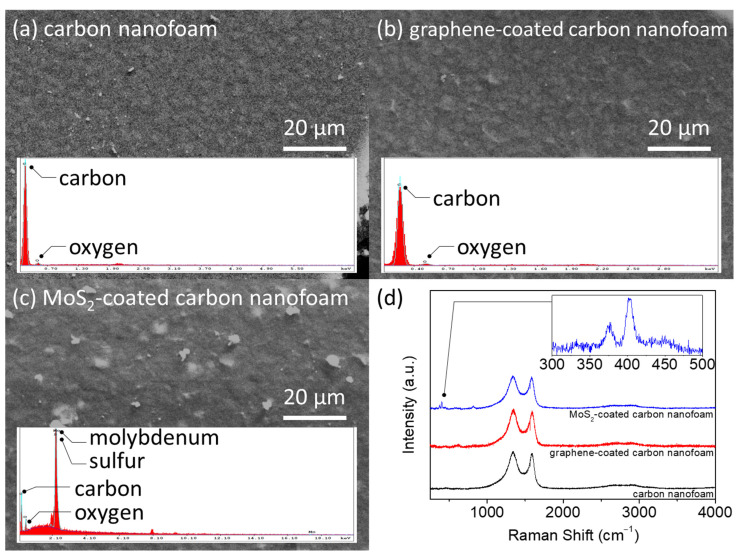
Chemical analysis: microstructural and elemental analyses of (**a**) carbon nanofoam, (**b**) graphene-coated carbon nanofoam, and (**c**) MoS_2_-coated carbon nanofoam. (**d**) Raman spectra.

Figure 2 presents the elemental and Raman analyses of the unmodified, graphene-coated, and MoS_2_-coated carbon nanofoams. The energy-dispersive X-ray spectroscopy (EDX) results show high-intensity elemental carbon signals in the unmodified and graphene-coated carbon nanofoams (Figure 2a,b). The MoS_2_-coated carbon nanofoam shows intense elemental molybdenum and sulfur signals owing to its coating (Figure 2c). Although the MoS_2_-coated carbon nanofoam has a different surface elemental composition, the top-view microstructural observation confirms the lack of significant physical changes in, or damage to, the samples.

Figure 2d shows the Raman spectra of the carbon nanofoams. The use of a carbon nanofoam as the substrate yields a strong D band at 1350 cm^−1^ and G band at 1580 cm^−1^, which reflect disorder in the sp^2^-hybridized carbon systems and stretching of the C–C bond in ordered graphitic materials, respectively. Another strong peak in the 2500–2800 cm^−1^ range corresponds to the graphitic sp^2^ material 2D band that results from inelastic scattering due to the graphene structure [17,18,19]. The MoS_2_-coated carbon nanofoam is characterized by additional characteristic peaks E^1^_2g_ and A_1g_ at 376 cm^−1^ and 403 cm^−1^, respectively, as shown in the inset. The in-plane E^1^_2g_ mode results from the vibration of sulfur atoms in one direction and of the molybdenum atom in the opposite direction, and the out-of-plane A_1g_ mode results from the out-of-plane vibration of the sulfur atoms [29,30,31]. We next examine the I_D_/I_G_ ratios of the unmodified, graphene-coated, and MoS_2_-coated carbon nanofoams, which are 1.66, 1.84, and 1.60, respectively. The relatively high ratio of the graphene-coated carbon nanofoam indicates the deposition of a layer of defective graphene on the carbon nanofoam.

Based on the material characterization summarized above, it is worth noting that the unique material properties of fast electron transfer and polysulfide adsorption of the carbon nanofoam, with its conductive and porous structure, are strengthened by the graphene coating and MoS_2_ coating, respectively. The prominent differences in porosity and conductivity between the unmodified and coated nanofoams make them suitable to be used as platforms for the analysis of the battery chemistry of lithium–sulfur systems.

### 3.2. Electrochemistry of the Sulfur Cathode with the Carbon Nanofoams

**Figure 3:**
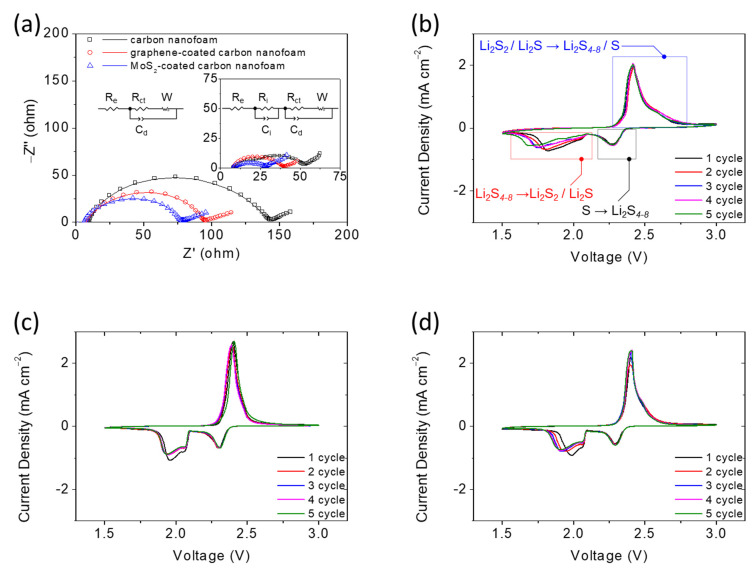
Electrochemical analysis: (**a**) electrochemical impedance analysis of the high-loading sulfur cathodes with various carbon nanofoams before and after cycling (inset). The data points are the experimental impedance data, and the data curves are the fitting impedance data obtained by the equivalent circuits. Cyclic voltammograms of sulfur cathodes with (**b**) the carbon nanofoam, (**c**) the graphene-coated carbon nanofoam, and (**d**) the MoS_2_-coated carbon nanofoam at 0.02 mV s^−1^.

Figure 3 shows a summary of the electrochemical analysis of the lithium–sulfur battery cathode with the different carbon nanofoams as advanced current collectors. The cathode electrochemistry is analyzed with a high-loading sulfur cathode that attains a sulfur loading of 4.8 mg cm^−2^ at a low electrolyte-to-sulfur ratio of 10 µL mg^−1^. This allows the investigation of the insulating nature of the solid-state active materials and the diffusion of the liquid-state active material, which often affect the electrochemical performance of the high-loading sulfur cathode [10,11,12,13].

Figure 3a presents the electrochemical impedance analysis of cells with different carbon nanofoams to explore the impedance/resistance before and after cycling (the inset). The Nyquist plots (i.e., the data points) with the corresponding equivalent circuits (i.e., the fitting curves) indicate a semicircle and a slash before cycling, which are associated with the ohmic resistance (Re) of the whole device, the charge transfer resistance (Rct) of the charge transfer kinetics, and Warburg impendence (W) of the lithium-ion diffusion, respectively [10,11,12,13]. The high-loading sulfur cathodes with the unmodified, graphene-coated, and MoS_2_-coated carbon nanofoams possess the Re and Rct values of 9.3 and 133.9, 7.9 and 87.4, and 7.7 and 71.3 Ω, respectively (Table S1, Supplementary Materials). The high-loading sulfur cathode with the carbon nanofoam has a low charge transfer resistance because the carbon nanofoam accommodates the large amount of active material within its conductive carbon matrix. The graphene and MoS_2_ coatings further reduce the resistance because the graphene coating improves the charge transfer of the cathode [10,17,18,19] and the MoS_2_ coating confers both high electron mobility and polysulfide stabilization [10,30,31,32]. These features result in low resistance, which could improve the reaction kinetics and electrochemical efficiency of the cathode. The inset shows the electrochemical impedance analysis of the cells after cycling, which depicts the interface resistance (Ri) that corresponds to the deposition of a solid insulation layer on the surface of the electrodes at the high frequency region [10,11,12,13]. The cycled sulfur cathodes with the unmodified, graphene-coated, and MoS_2_-coated carbon nanofoams possess the Re, Ri, and Rct values of 8.1, 9.3, and 36.6, 7.8, 8.9, and 23.3, and 7.6, 9.1, and 11.3 Ω, respectively (Table S1, Supplementary Materials). As a reference, the interface capacitance (Ci) and the double-layer capacitance (Cd) are the constant phase elements. The impedance semicircles of the cathode with different carbon nanofoams decrease because the carbon nanofoams serve as a conductive network, trap the active material, and reactivate the trapped active material during cycling. This limits the formation of insulating active material agglomerates while retaining the dissolved polysulfides within the cathode region as the catholyte [12,26,27,28]. The graphene-coated and MoS_2_-coated carbon nanofoams exhibit even lower cathode resistance as the coatings confer high conductance and polysulfide trapping, respectively. Thus, the electrochemical impedance results demonstrate that the carbon nanofoam current collectors endow the high-loading sulfur cathode with low cathode resistance and high utilization of the trapped active material. 

Figure 3b–d show the cyclic voltammograms of the unmodified, graphene-coated, and MoS_2_-coated carbon nanofoams, respectively. The cyclic voltammograms of all three types of carbon nanofoams have similar shapes; each has a pair of cathodic peaks and a continuous anodic peak. The two cathodic peaks reflect the reduction conversion from solid-state sulfur to liquid-state polysulfides at ~2.4 V (cathodic-1 peak) and then to solid-state sulfide mixtures (Li_2_S_2_/Li_2_S) at ~2.1V (cathodic-2 peak) [5,9,10,11]. The continuous anodic peaks at ~2.3 V (anodic peak) correspond to the reversible oxidation from solid-state sulfides to liquid-state polysulfides and solid-state sulfur [5,9,10,11]. The high-loading sulfur cathode with the carbon nanofoam shows almost unchanged cathodic-1 and anodic peaks, indicating the excellent polysulfide retention and enhanced redox reaction. However, the overlapping cathodic-2 peak shows a slight shift of the peak current density toward the low voltage region, which implies a slight increase in the polarization of the high-loading sulfur cathode in the lean-electrolyte lithium–sulfur batteries; however, the slight increase in the polarization would not affect the electrochemical reversibility of the cell. On the other hand, notably, repeated scans of the graphene-coated and MoS_2_-coated carbon nanofoams do not reveal any apparent decreases in current or shifts in potential in these reduction and oxidation peaks. The overlapping redox curves attest to the superior cell reversibility and stability contributed by the coated nanofoams [9,10,11]. The improved electrochemical reaction of the graphene-coated carbon nanofoam mainly arises from the conductive graphene coating, which amplifies the fast charge transfer of the matrix. Thus, the sluggish reduction reaction from polysulfide to sulfide is ameliorated, which maintain the reduction peaks as almost unchanged; the solid-state active material maintains the high reutilization during cycling [10,17,18,19]. The enhanced redox reaction of the MoS_2_-coated carbon nanofoam could be attributed to the MoS_2_ coating, which confers a strong polysulfide-trapping capability and fast electron mobility. The resulting strong polysulfide retention and high reaction kinetics are characterized by the overlapping reduction and oxidation peaks [10,30,31,32]. Thus, electrochemical analysis of the carbon nanofoams indicates that the use of the carbon nanofoam as a porous current collector would allow the development of a high-performance sulfur cathode with a high sulfur loading of 4.8 mg cm^−2^ and high electrochemical reversibility at the low electrolyte-to-sulfur ratio of 10 µL mg^−1^. Moreover, optimally coated carbon nanofoams could boost the electrochemical performance with high utilization and stability.

**Figure 4:**
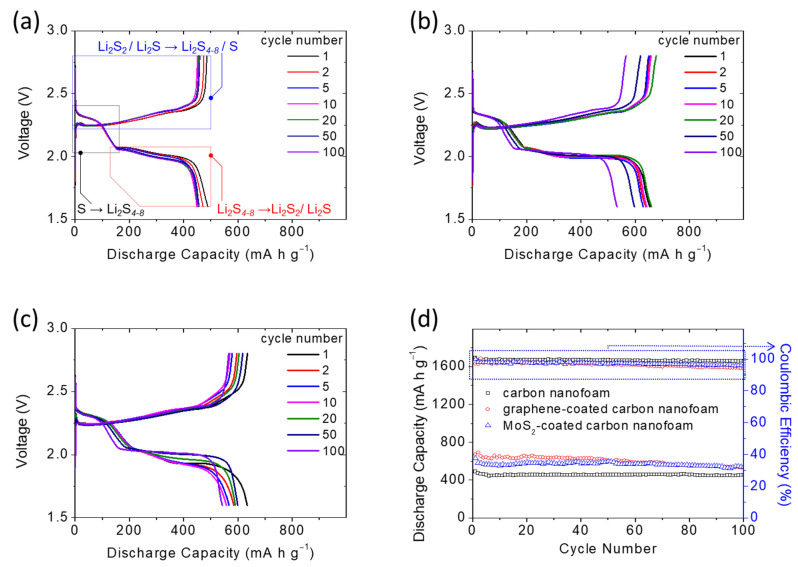
Electrochemical performance: discharge/charge voltage profiles of sulfur cathodes with (**a**) the carbon nanofoam, (**b**) the graphene-coated carbon nanofoam, and (**c**) the MoS_2_-coated carbon nanofoam. (**d**) Cyclability at the C/10 rate.

Figure 4a–c present the discharge/charge voltage profiles of cells with different carbon nanofoams over 100 cycles. The cell with the unmodified carbon nanofoam (Figure 4a) displays two distinguishable discharge plateaus and a continuous charge plateau, consistent with the cyclic voltammograms. During discharge, the two separate discharge plateaus indicate the two reduction reactions: the reduction from sulfur to polysulfides at ~2.4 V and the subsequent reduction from polysulfides to sulfides at ~2.1 V [5,9,10,11]. The overlapping upper discharge curves indicate the advantages of using the carbon nanofoam to host the large amount of active material and inhibit the fast polysulfide diffusion. The lower discharge plateaus are well-retained during cycling, representing the improved reaction kinetics of the cathode that has a large amount of insulating sulfur and a low amount of electrolyte. Moreover, the carbon nanofoam continuously transfers electrons and channels electrolyte to reactivate the trapped active material, facilitating the stable cell cycling with high retention. During charging, the two continuous plateaus at ~2.3 V could be attributed to the reversible oxidation reactions of sulfide to polysulfides and sulfur [9,10,11]. Figure 4b,c show the discharge/charge voltage profiles of cells with the graphene-coated and MoS_2_-coated carbon nanofoams, respectively. The high-conductivity graphene coating confers fast charge transfer capabilities to the active material in the cathode, which is trapped in the carbon nanofoam. This facilitates the electrochemical utilization of sulfur (Figure 4b). In addition to enhanced conductivity, the MoS_2_-coated carbon nanofoam allows the original carbon nanofoam to physically trap the diffusing polysulfides and chemically adsorb them, further improving the electrochemical stability during cell discharge and charge (Figure 4c).

Figure 4d shows the cyclability of the high-loading sulfur cathode with the carbon nanofoam current collectors. The cycling performance demonstrates that the carbon nanofoam current collector significantly enhances the electrochemical stability and efficiency of the high-loading sulfur cathode in the lean-electrolyte lithium–sulfur cell. The graphene-coated and MoS_2_-coated carbon nanofoams further improve the electrochemical utilization of the large amount of sulfur. The high-loading sulfur cathodes with the unmodified, graphene-coated, and MoS_2_-coated carbon nanofoams attain high charge storage capacities of 490, 672, and 633 mA·h g^−^^1^, respectively, at the C/10 rate. After 100 cycles, the reversible capacities remain as high as 452, 532, and 548 mA·h g^−^^1^ at the C/10 rate, respectively, corresponding to high capacity retentions of 92, 79, and 87%, respectively. This superior cycling stability suggests that the carbon nanofoam current collectors provide a more stable electrochemical environment for the electrochemical conversion-type sulfur cathode than conventional cells, which are optimal cathode configuration for high-loading sulfur cathodes.

**Figure 5:**
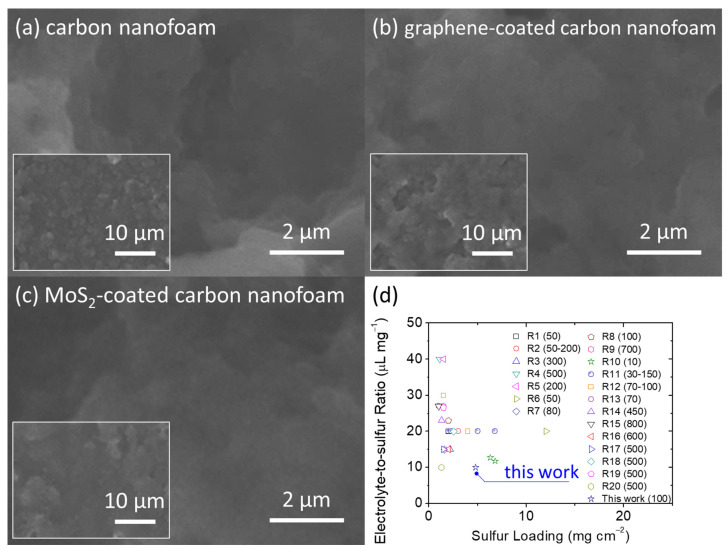
Battery performance: microstructural inspection of (**a**) carbon nanofoam, (**b**) graphene-coated carbon nanofoam, and (**c**) MoS_2_-coated carbon nanofoam retrieved from cycled cathodes after 100 cycles. (**d**) Comparison with literature data: sulfur loading, electrolyte-to-sulfur ratio, and the cycle life in the parentheses.

After the electrochemical analysis, we retrieved the high-loading sulfur cathodes from the cycled cells after 100 cycles at discharge status. Figure 5a–c show the SEM inspection of the carbon nanofoams of the cycled cathodes. The microstructural images depict the distinguishable retention of the active material, with no obvious agglomeration of the insulating solid-state active materials after cycling. This positive feature confirms the good encapsulation and stabilization of the active material in the cathode [26,27,28]. Moreover, the battery performance depicts the research trend in developing high-loading sulfur cathodes in the lean-electrolyte lithium–sulfur cells with enhanced electrochemical stability (Figure 5d and Table S2, Supplementary Materials). The analytical results indicate the necessity in designing and analyzing the high-loading sulfur cathode in the cell with low amounts of electrolyte featuring a low electrolyte-to-sulfur ratio while maintaining the necessary cycle stability for realizing long cycle life with high capacity retention [10,11,12,13,14,15,16]. Among the published lithium–sulfur studies that report on the sulfur loading and electrolyte-to-sulfur ratio, our carbon nanofoam simultaneously attains high sulfur loading and a low electrolyte-to-sulfur ratio while attaining the necessary cycle life featuring the highest capacity retention. Therefore, the comparison analysis confirms the feasibility of adopting the carbon nanofoams in designing high-sulfur-loading cathodes with high electrochemical performance at a low electrolyte condition.

The high electrochemical stability of the carbon nanofoams confirms that the inclusion of conductive and porous current collectors improves the cycle stability by hosting the insulating active material in the cathode substrate and stabilizing the diffusing polysulfides within the cathode region. The conductive skeleton and porous network of the carbon nanofoam confer the trapped active material with fast electron transfer and smooth electrolyte diffusion capabilities, resulting in superior retention of the active material and higher capacity [12,13,14,26,27,28]. The graphene-coated carbon nanofoam attains the highest peak charge storage capacity because the conductive graphene coating improves the conductivity of the cathode, which further improves the reaction kinetics and electrochemical utilization [17,18,19]. The MoS_2_-coated carbon nanofoam also shows good conductivity and further possesses a strong polysulfide-trapping capability owing to the MoS_2_ coating, which improves the electrochemical utilization and stability of the high-loading sulfur cathode [30,31,32].

## 4. Conclusions

In summary, the carbon nanofoam current collector offers a practical option for the development of a high-performance sulfur cathode with high sulfur loading and enhanced electrochemical stability in a lean-electrolyte lithium–sulfur cell. The improvements in the overall cathode parameters and performance arise from the conductive and porous structure of the carbon nanofoam, which hastens the sluggish conversion reaction between the solid-state and liquid-state active materials, encapsulates a large amount of sulfur, and traps the migrating polysulfides within the cathode as catholytes. The modified graphene-coated and MoS_2_-coated carbon nanofoams further amplify the desirable material properties of carbon nanofoams by virtue of the conductive graphene and polysulfide-trapping MoS_2_ coatings, respectively. The high-loading sulfur cathodes with graphene-coated and MoS_2_-coated carbon nanofoams exhibit high charge -storage capacities of 672 and 633 mA·h g^−^^1^, respectively, with a superior cycle stability and high capacity retention of 79–87% after 100 cycles.

## Supplementary Materials

The following are available online at https://www.mdpi.com/article/10.3390/nano11082083/s1, Table S1: Electrochemical impedance analysis of the high-loading sulfur cathodes with various carbon nanofoams before and after cycling, Table S2: Comparative analysis of the battery performances and electrochemical characteristics of the sulfur cathodes in the lithium–sulfur research, supporting references: R1–R20.
