# Supplementary material for: Advanced Current Collectors with Carbon Nanofoams for Electrochemically Stable Lithium—Sulfur Cells
Source: Nanomaterials (Basel). 2021 Aug 17;11(8):2083. doi: 10.3390/nano11082083 (PMC8398066; doi:10.3390/nano11082083)
Supplement: Supplementary file 1 [file nanomaterials-11-02083-s001.zip › nanomaterials-1334193-supplementary.pdf]

nanomaterials

MDPI

Supplementary Materials

Advanced Current Collectors with Carbon Nanofoams for Electrochemically Stable Lithium–Sulfur Cells

Shu-Yu Chen [1] and Sheng-Heng Chung [1,2,*]

[1] Department of Materials Science and Engineering, National Cheng Kung University, No.1, University Road, Tainan City 701, Taiwan; f64071203@gs.ncku.edu.tw
[2] Hierarchical Green-Energy Materials Research Center, National Cheng Kung University, No.1, University Road, Tainan City 701, Taiwan
* Correspondence: SHChung@gs.ncku.edu.tw

Table S1. Electrochemical impedance analysis of the high-loading sulfur cathodes with various carbon nanofoams before and after cycling.

testing conditions	testing samples	ohmic resistance (Re, Ω)	interface resistance (Ri, Ω)	charge-transfer resistance (Rct, Ω)
before cycling	carbon nanofoam	9.3	-	133.9
before cycling	graphene-coated carbon nanofoam	7.9	-	87.4
before cycling	MoS_2-coated carbon nanofoam	7.7	-	71.3
after 100 cycles	carbon nanofoams	8.1	9.3	36.6
after 100 cycles	graphene-coated carbon nanofoam	7.8	8.9	23.3
after 100 cycles	MoS_2-coated carbon nanofoam	7.6	9.1	11.3

Table S2. Comparative analysis of the battery performances and electrochemical characteristics of the sulfur cathodes in the lithium–sulfur research.

sulfur loading (mg cm^{-2})	electrolyte-to-sulfur ratio (μL mg^{-1})	peak capacity (mA·h g^{-1})	reversible capacity (mA·h g^{-1})	capacity retention (%)	cycle life	rate	ref.
2	20	888	810	91	50	C/5	R1
2	20	750	720	96	50	C/5	R1
3	20	1,085	1,031	95	50	C/5	R2
3	20	867	776	90	200	1C	R2
2.2	15	1,270	917	73	300	C/5	R3
1.1	40	540	413	77	500	5C	R4
1.5	40	1,499	808	54	200	C/5	R5
12	20	1,126	799	71	50	C/5	R6
1.6	15	1,107	1,034	93	80	C/5	R7
2	23	1,137	1,030	91	100	C/2	R8
1.5	27	834	367	44	700	2/3C	R9
6.8	12	1,000	882	88	10	C/20	R10
6.3	13	1,100	952	87	10	C/20	R10
2	20	1,104	954	86	100	C/5	R11
2.3	20	965	696	72	150	C/10	R11

5	20	1,104	800	72	80	C/10	R11
6.8	20	1,387	956	69	30	C/10	R11
1.5	30	1,471	715	49	100	C/2	R12
4	20	1,000	700	70	70	C/5	R12
1	27	1,661	1,342	81	70	C/5	R13
1.3	23	950	480	51	450	C/5	R14
1	27	752	520	69	800	1C	R15
2	15	1,098	656	60	600	C/2	R16
1.5	15	900	450	50	500	1C	R17
2.54	20	1,125	696	62	500	C/5	R18
1.5	27	913	586	64	500	1C	R19
1.3	10	917	577	63	500	1C	R20
4.8	10	490	452	92	100	C/10	This work
4.8	10	672	532	79	100	C/10	This work
4.8	10	633	548	86	100	C/10	This work

References

R1. Chung, S.-H.; Manthiram, A. Lithium–sulfur batteries with superior cycle stability by employing porous current collectors. *Electrochim. Acta* **2013**, *107*, 569-576.

R2. Zhao, T.; Ye, Y.; Peng, X.; Divitini, G.; Kim, H.-K.; Lao, C.-Y.; Coxon, P. R.; Xi, K.; Liu, Y.; Ducati, C.; Chen, R.; Kumar. R. V. Advanced lithium–sulfur batteries enabled by a bio-inspired polysulfide adsorptive brush. *Adv. Funct. Mater.* **2016**, *26*, 8418-8426.

R3. Lee, J. S.; Jun, J.; Jang, J.; Manthiram, A. Sulfur-immobilized, activated porous carbon nanotube composite based cathodes for lithium–sulfur batteries. *Small* **2017**, *13*, 1602984.

R4. Song, X.; Wang, S.; Bao, Y.; Liu, G.; Sun, W.; Ding, L.-X.; Liu, H.; Wang, H. A high strength, free-standing cathode constructed by regulating graphitization and the pore structure in nitrogen-doped carbon nanofibers for flexible lithium-sulfur batteries. *J. Mater. Chem. A* **2017**, *5*, 6832-6839.

R5. Li, Y.; Zhu, J.; Zhu, P.; Yan, C.; Jia, H.; Kiyak, Y.; Zang, J.; He, J.; Dirican, M.; Zhang, X. Glass fiber separator coated by porous carbon nanofiber derived from immiscible PAN/PMMA for high-performance lithium-sulfur batteries. *J. Membr. Sci.* **2018**, *552*, 31-42.

R6. Zhang, Y.-Z.; Zhang, Z.; Liu, S.; Li, G.-R.; Gao, X.-P. Free-standing porous carbon nanofiber/carbon nanotube film as sulfur immobilizer with high areal capacity for lithium-sulfur battery. *ACS Appl. Mater. Interfaces* **2018**, *10*, 8749-8757.

R7. Wang, M.; Song, Y.; Sun, Z.; Shao, Y.; Wei, C.; Xia, Z.; Tian, Z.; Liu, Z.; Sun, J. Conductive and catalytic VTe_2@MgO heterostructure as effective polysulfide promotor for lithium-sulfur batteries. *ACS Nano* **2019**, *13*, 13235-13243.

R8. Zhang, H.; Zhao, Z.; Hou, Y.-N.; Tang, Y.; Liang, J.; Liu, X.; Zhang, Z.; Wang, X.; Qiu, J. Highly stable lithium–sulfur batteries based on p–n heterojunctions embedded on hollow sheath carbon propelling polysulfides conversion. *J. Mater. Chem. A* **2019**, *7*, 9230-9240.

R9. Yue, Z.; Dunya, H.; Kucuk, K.; Aryal, S.; Ma, Q.; Antonov, S.; Ashuri, M.; Alabbad, B.; Lin, Y.; Segre, C. U. MnO_2-coated sulfur-filled hollow carbon nanosphere-based cathode materials for enhancing electrochemical performance of Li-S cells. *J. Electrochem. Soc.* **2019,** *166*, A1355.

R10. Marangon, V.; Di Lecce, D.; Orsatti, F.; Brett, D. J.; Shearing, P. R.; Hassoun, J. Investigating high-performance sulfur–metal nanocomposites for lithium batteries. *Sustainable Energy Fuels* **2020,** *4*, 2907-2923.

R11. Ji, J.; Sha, Y.; Li, Z.; Gao, X.; Zhang, T.; Zhou, S.; Qiu, T.; Zhou, S.; Zhang, L.; Ling, M. Selective adsorption and electrocatalysis of polysulfides through hexatomic nickel clusters embedded in N-doped graphene toward high-performance Li-S batteries. *Research* **2020**, *2020*, 5714349.

R12. Wei, Y.; Wang, Y.; Zhang, X.; Wang, B.; Wang, Q.; Wu, N.; Zhang, Y.; Wu, H. Superhierarchical conductive framework implanted with nickel/graphitic carbon nanocages as sulfur/lithium metal dual-role hosts for Li–S batteries. *ACS Appl. Mater. Interfaces* **2020,** *12*, 35058-35070.

R13. Dunya, H.; Ashuri, M.; Alramahi, D.; Yue, Z.; Kucuk, K.; Segre, C. U.; Mandal, B. K. MnO_2-coated dual core–shell spindle-like nanorods for improved capacity retention of lithium–sulfur batteries. *ChemEngineering* **2020,** *4*, 42.

R14. Rafie, A.; Singh, A.; Kalra, V. Synergistic effect of sulfur-rich copolymer/S_8 and carbon host porosity in Li-S batteries. *Electrochim. Acta* **2021,** *365*, 137088.

R15. Dunya, H.; Ashuri, M.; Yue, Z.; Kucuk, K.; Lin, Y.; Alramahi, D.; Segre, C. U.; Mandal, B. K. Rational design of titanium oxide-coated dual core–shell sulfur nanocomposite cathode for highly stable lithium–sulfur batteries. *J. Phys. Chem. Solids* **2021,** *149*, 109791.

R16. Li, Q.; Liu, Y.; Yang, L.; Wang, Y.; Liu, Y.; Chen, Y.; Guo, X.; Wu, Z.; Zhong, B. N, O co-doped chlorella-based biomass carbon modified separator for lithium-sulfur battery with high capacity and long cycle performance. *J. Colloid Interface Sci.* **2021,** *585*, 43-50.

R17. Long, B.; Ma, J.; Song, T.; Liu, L.; Wang, X.; Song, S.; Tong, Y. Bifunctional polyvinylpyrrolidone generates sulfur-rich copolymer and acts as "residence" of polysulfide for advanced lithium-sulfur battery. *Chem. Eng. J.* **2021,** *414*, 128799.

R18. Park, J. H.; Choi, W. Y.; Yang, J.; Kim, D.; Gim, H.; Lee, J. W. Nitrogen-rich hierarchical porous carbon paper for a free-standing cathode of lithium sulfur battery. *Carbon* **2021,** *172*, 624-636.

R19. Zheng, S.; Sun, D.; Wu, L.; Liu, S.; Liu, G. Carbon fiber supported two-dimensional ZIF-7 interlayer for durable lithium-sulfur battery. *J. Alloys Compd.* **2021,** *870*, 159412.

R20. Jin, J.; Cai, J.; Wang, X.; Sun, Z.; Song, Y.; Sun, J. Architecturing aligned orthorhombic Nb_2O_5 nanowires toward sodium-ion hybrid capacitor and lithium–sulfur battery applications. *FlatChem* **2021,** *27*, 100236.
